# Activities of Daily Living Disability Transition Patterns in Older Adults with Chronic Diseases: A Four-Year Cohort Study in China

**DOI:** 10.3390/healthcare12202088

**Published:** 2024-10-20

**Authors:** Tian Guo, Yunwei Zhang, Gang Xu, Wenxian Liu, Hansheng Ding, Shaofan Chen

**Affiliations:** 1 School of Health Policy and Management, Nanjing Medical University, No. 101 Longmian Avenue, Nanjing 211166, China; tianguo@stu.njmu.edu.cn (T.G.); liuwenxian29@163.com (W.L.); 2 Jiangsu Provincial Institute of Health, Nanjing Medical University, No. 101 Longmian Avenue, Nanjing 211166, China; 3 Shanghai Health Development Research Center (Shanghai Medical Information Center), No. 602 Jianguo (W) Road, Xuhui District, Shanghai 200031, China; yunwei1202@126.com; 4 School of Public Health, Shanghai Jiaotong University, No. 227 South Chongqing Road, Shanghai 200025, China; gangxu@sjtu.edu.cn

**Keywords:** older adults, chronic diseases, ADL disability, multi-state Markov model, transition

## Abstract

Background: Older adults with chronic diseases often experience higher rates of Activities of Daily Living (ADL) disability, with research primarily examining the transition between states of ADL disability and non-disability. The current study aims to analyze the patterns and factors of mutual transitions between multiple different ADL disability states in older adults with chronic diseases. Methods: This longitudinal study utilized data from the Shanghai Elderly Care Unified Needs Assessment (SECUNA) spanning 2014 to 2017, with 2014 being the baseline. The study included older adults aged 60 years and older with chronic diseases. Using the Markov model, individuals were classified into three states: no ADL disability, mild ADL disability, and severe ADL disability. Transition patterns were analyzed by calculating the frequency, intensity, and probability of transition, and the influencing factors of six transition scenarios were evaluated. Results: Older adults with mild ADL disability were more likely to experience improvement (transition intensity: 0.4731) rather than deterioration (transition intensity: 0.2226) in their ADL disability states. However, those with severe ADL disability faced challenges in improving their states (transition intensities: 0.0068 and 0.1204). Among the six ADL disability transition scenarios, place of residence was associated with four scenarios, age and economic sources were associated with three scenarios, sex was associated with two scenarios, and other factors were associated with one scenario. Conclusions: The transition patterns and factors differ among individuals with varying ADL disability states. It is essential for relevant agencies to implement tailored preventive healthcare strategies to effectively manage the health status of older adults with chronic diseases.

## 1. Introduction

China has the world’s largest older population and is one of the fastest-aging countries [1]. Aging is associated with significantly increased vulnerability in both physical and psychological health, making elderly individuals highly susceptible to various diseases [2]. *The Healthy China Action 2019–2030* illustrated that 180 million older adults in China have chronic diseases, accounting for 75% of the older population in China [3]. Deaths from chronic diseases occupy 86.6% of total deaths in China, while disease burden (the pressure on the overall socio-economic and health of society) exceeds 70% of the total burden [4]. Therefore, it is imperative that the health status of older adults suffering from chronic conditions receive substantial attention.

‘Older population’ has a different definition in different countries. In China, it usually refers to residents who are older than 60 years old [3]. Activities of Daily Living (ADL), an important indicator for appraising the health status of older adults, are instrumental in evaluating the deterioration of daily physical functions [5]. ADL include using restrooms, eating, dressing, grooming, walking, and bathing. An ADL disability denotes an individual’s incapacity to autonomously perform fundamental tasks, resulting in repercussions for the individual and their family and society at large [6]. Studies reported that chronic diseases heighten the likelihood of ADL impairment in older adults, attributable to characteristics such as protracted illness duration, gradual recuperation, heightened vulnerability to complications, and the presence of potential risk factors associated with functional disability. As a result, older adults with chronic diseases have a higher prevalence of ADL disability [7,8,9]. ADL disability transition refers to a change in an individual’s incapacity to autonomously perform fundamental tasks, which may involve the worsening, recovery, or improvement of ADL.

A number of studies analyzed the patterns of ADL disability transitions, while most of them focused on the situation between two states (with and without ADL disability) [10,11,12]. However, the occurrence of ADL disability is a dynamic and progressive process [13]. The multi-state Markov model, a tool for dealing with longitudinal data, is capable of the continuous dynamic of stochastic processes, including the transitions among multiple different ADL disability states [14]. Furthermore, it can simultaneously consider all states, outcomes, and timing of transitions between states and dynamically evaluate the progression of diseases [15]. Various factors impact ADL disability incidence, such as sex, age, institutional contexts, education, marital status, and social activities [13]. However, limited research exists on factors affecting transitions between ADL disability states.

The current study constructed a multi-state Markov model and adopted a retrospective cohort study design to analyze the patterns and factors of mutual transitions between multiple different ADL disability states in older adults with chronic diseases.

## 2. Methods

### 2.1. Study Population

Shanghai Elderly Care Unified Needs Assessment (SECUNA) dataset was used in the present study. SECUNA is a longitudinal follow-up conducted in Shanghai (China) from 2013 to 2021. SECUNA was designed to investigate the long-term care needs of older adults, including those in the community and various institutions. The current study used 2014, 2015, 2016, and 2017 data, with 2014 as the baseline. The inclusion criteria included (1) age ≥ 60 years old; (2) chronic diseases throughout 4 years (both new and pre-existing cases included); (3) investigated by SECUNA at least twice. The exclusion criteria were (1) deceased participants and (2) missing data on chronic diseases, ADL, or any covariates.

### 2.2. Outcome Measurement

ADL was assessed with a 20-item self-design scale, totaling 20 points (See Appendix A, Table A1), adapted from Lawton and Brody’s 1969 scale [16]. The points for each item ranged from 0 to 1. Points from the 20 items were summed to calculate the participants’ total ADL points. A point of 20 being no ADL disability, a point range from 8 to 20 (excluding 20) being mild ADL disability, and a point range from 0 to 8 (excluding 8) being severe ADL disability.

### 2.3. Chronic Disease Situation

SECUNA listed ten chronic diseases: diabetes, hypertension, chronic obstructive pulmonary disease, chronic pneumonia, Parkinson’s disease, cerebral hemorrhage, advanced tumors, coronary atherosclerotic heart disease, cerebral infarction, and fractures of the lower limbs. Investigators assessed each condition, confirmed by family doctors and medical records for quality control.

### 2.4. Covariates

We used covariates to determine which factors influenced transitions between different ADL disability states in older adults with chronic diseases. The socio-demographic information included age, sex, and educational level. The educational level was classified as illiteracy, primary school, middle school, high school, and college or higher level.

The place of residence was divided into two categories: living at home (including freehold houses, private houses, and various types of rented houses) and institutions for older adults (including nursing homes, care and attention homes, community health service centers, and hospitals). The economic sources were classified by the existence of a stable pension (with pensions; /without pensions and need for family, friends, or other allowances). The co-morbidity group included older patients with two or more chronic diseases.

### 2.5. Statistical Analysis

Participant baseline characteristics were described using frequency and composition ratios. The multi-state Markov model in this study comprised three interconvertible states: no ADL disability, mild ADL disability, and severe ADL disability, with no absorbing state due to a low mortality rate. An absorbing state describes a state where, once you enter it, you cannot leave. It is a way to model situations where something reaches an end state and cannot go back to how it was before. In the current study, the absorbing state means death.

Following the calculation of ADL disability state transition frequency in older adults with chronic disease over four years, we estimated transition intensity ($q_{rs}$) between states and the one-year transition probability. Transition intensity measures the speed at which things change from state to state in a Markov model. The higher the intensity, the faster the transition can happen, and the lower it is, the slower the transition will be. $q_{rs}$ describes the instantaneous risk of moving from state r to state s at time t, while the one-year transition probability represents the likelihood that an individual in state *r* at time *t* will be in state s one year later.

$$q_{rs}=\underset{∆t\to 0}{\lim}\frac{p\left(s\left(t+∆t\right)=s|S\left(t\right)=r\right)}{∆t}$$

In addition, the effects of covariates on a particular transition intensity were estimated by modeling the intensity as a function of these variables, similar to a proportional hazards model [17]. Proportional hazards model takes survival outcome and survival time as dependent variables and can simultaneously analyze the impact of numerous factors on survival period [18].

qrszt=qrs0expβztrsT

We estimated the effects of covariates on six transition scenarios, with age modeled as a continuous variable and education level as an ordered categorical variable. Meanwhile, sex, place of residence, economic source, and chronic disease grouping are grouped as unordered categorical variables. A detailed explanation of the model has been reported in previous studies [17,19].

Descriptive statistics of baseline characteristics were conducted using IBM SPSS 25.0 [20]. The multi-state Markov model was developed using the MSM package in R 4.3.2 [19]. The plot function of R 4.3.2 was utilized to illustrate actual and theoretical frequency percentage curves for each state, evaluating the fit of the multi-state Markov model [21].

## 3. Results

### 3.1. Socio-Demographic Characteristics at Baseline

The study included 9091 older adults with chronic diseases, average age (77.87 ± 9.22) years. Among socio-demographic characteristics, 41.6% were male, 60.1% were home-based, and 51.6% had co-morbid chronic diseases. At baseline, 51.2% had no ADL disability, while 26.8% and 22.0% had mild and severe ADL disability, respectively. Factors associated with ADL disability included older age, female gender, lower education, institutional residence, reliance on support, and comorbidity (see Table 1).

### 3.2. ADL Disability State Transition in Older Adults with Chronic Diseases

Table 2 illustrates the frequency of ADL disability state transition in older adults with chronic diseases. Older adults with no ADL disability tended to keep their state unchanged. Meanwhile, the frequency of their transitions to severe ADL disability was extremely low, far lower than in the other two situations. Although older adults with mild ADL disability tended to maintain their conditions, the difference in the transitions to no ADL disability was not significant. Older adults with severe ADL disability remained largely unchanged in their state, with very few transitions to no ADL disability and mild ADL disability.

Table 2 also demonstrates the transition intensity from one ADL condition to another. The transition intensity on the main diagonal of the matrix represents the rate at which the system leaves a particular state. This rate is equal to the negative sum of the transition intensities in the same row but off the diagonal, which represents the rates of moving to other states. Older adults with no ADL disability were most likely to transition to mild ADL disability in a very short time if they experienced state transitions. Its transition intensity was much higher than that of the transition to severe ADL disability. The transition intensity of older adults with mild ADL disability to no ADL disability was approximately twice as high as the transition intensity of older adults with mild ADL disability to severe ADL disability. Those with severe ADL disability were most likely to transition to mild ADL disability if they experienced a state transition. Its transition intensity was 17.71 times higher than the transition intensity of older adults with severe ADL disability to no ADL disability. From the above analysis, it can be seen that the ADL disability status has the potential to be improved. Mild ADL disability, which has the highest intensity of improvement (0.4731), might be the best time for receiving treatments or interventions.

Table 2 illustrates the one-year transition probability. The transition probability for each ADL disability state was mainly concentrated near the main diagonal, with probabilities below the main diagonal indicating improvement in state and probabilities above the main diagonal indicating deterioration in state. The probability of keeping their states unchanged showed a decreasing and then increasing trend from no ADL disability to severe ADL disability. Older adults with mild ADL disability had the highest probability of state transition, while those with severe ADL disability had the lowest probability of state transition. Older adults with no ADL disability had a probability of 0.8022 in keeping their states unchanged. Meanwhile, their probability of transition to mild ADL disability was higher than that to severe ADL disability. The probability of state improvement in older adults with mild ADL disability was about twice that of state deterioration. Older adults with severe ADL disability were more likely to transition to mild ADL disability than to no ADL disability. Similar to the transition intensity, the probability of state improvement decreased as the degree of ADL disability increased in older adults with chronic diseases.

### 3.3. Analysis of Factors Influencing ADL Disability State Transition in Older Adults with Chronic Diseases

The variables included were age, sex, educational level, place of residence, economic sources, and chronic disease grouping. The results of the univariate analysis showed that all variables had an impact on the transition of ADL disability in older adults with chronic diseases (see Appendix A, Table A2). Then, these variables were included in the multivariate analysis, and the results showed that the above variables still affected the transition of ADL disability in older adults with chronic diseases. However, the transition scenarios they affected were not the same (See Table 3).

As age increased, the transition intensity of state deterioration in older adults with no ADL disability increased, and the transition intensity of state improvement in older adults with mild ADL disability decreased. Compared to males, females had a lower transition intensity of state deterioration from mild ADL disability but also a lower transition intensity of state improvement from severe ADL disability (3-2). The influence of educational level was currently only reflected in the transition from mild ADL disability to severe ADL disability. As educational level increased, the transition intensity of state deterioration in older adults with mild ADL disability decreased. Compared with home-based older adults, older adults in institutions had a lower intensity of transition from no ADL disability to mild ADL disability but a higher intensity of transition from mild ADL disability to severe ADL disability. In addition, the intensity of state improvement for older adults with severe ADL disability in institutions was extremely low. The transition intensity of older adults who need allowances from no ADL disability to severe ADL disability was 4.65 times higher than that of older adults with pensions. Compared with older adults with pensions, older adults who need allowances had a lower intensity of state improvement from mild ADL disability but a higher intensity of state improvement from severe ADL disabilities (3-2). Compared with the non-co-morbidity group, co-morbidity older adults had a lower intensity of improvement from mild ADL disability, while others were not significant.

### 3.4. Model Assessment

**Figure 1:**
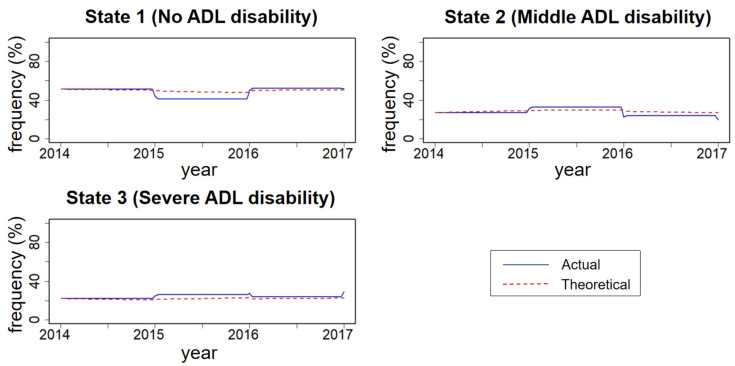
Evaluation plots for the multi-state Markov model.

The actual frequency percentage curves and the theoretical frequency percentage curves for each state were plotted over time. Figure 1 shows that the two curves of the actual and theoretical frequency percentages tended to coincide, indicating a good fit for the model.

Evaluation plots for the multi-state Markov model show the actual and theoretical frequency percentages for each state over time. State 1 represents no ADL disability, state 2 represents mild ADL disability, and state 3 represents severe ADL disability.

## 4. Discussion

The study investigated transition patterns between three ADL disability states in older adults with chronic diseases. Those with mild ADL disability were more likely to experience improvement rather than deterioration, while those with severe ADL disability were more likely to remain where they were than show improvement. Among six transition scenarios, place of residence was associated with four, age and economic sources with three, and sex with two, and educational level and chronic diseases grouping were associated with one transition scenario.

Based on transition frequency, estimated intensity, and predicted probability, transitions in ADL disability states (improvement, non-change, or worsening) are consistently observed in older adults with chronic diseases, aligning with prior research [22,23,24]. This suggests that the development of disability in old age is not unidirectional—it can both gradually deteriorate and potentially improve over time. Hence, nursing interventions and other strategies should aim to improve ADL states and enhance older adults’ quality of life. Studies have demonstrated the feasibility of strengthening exercise, nutrition, cognition, and social support [25,26,27,28,29].

However, among older adults with chronic diseases, state transitions were not the same for those with different ADL disability states, which makes nursing interventions more complex. In terms of transition intensity and one-year transition probability, older adults with mild ADL disability are more likely to improve rather than deteriorate. Proactive measures are needed to enhance support for maximizing positive change. Similar to a previous study [30], older adults with severe ADL disability face challenges in improving their state. They need comprehensive medical care, social support, and family care to cope with the mixed pressures of chronic diseases and disability. Older adults without ADL disability, although less likely to experience worsening, still have a probability of transitioning to severe disability within one year, according to the transition probability. This highlights the need for early prevention and regular health monitoring. Medical facilities and nursing homes should implement targeted preventive healthcare strategies to manage the health of older adults with chronic diseases effectively, focusing on improving their quality of life and reducing unnecessary medical costs.

The present study tested the impacts of several factors on the transition of ADL disability state in older adults with chronic diseases. Consistent with previous studies [31,32], increasing age correlated with decreased physical function in older adults, elevated risk of transitioning to mild and severe ADL disability, and reduced likelihood of improvement in ADL disability. Prior research indicated males were more likely to improve from ADL disability than females [33,34]. In our study, this trend was evident transitioning from severe to mild disability only. Another study indicated that males had higher rates of improvement in ADL disability than females at ages 50–65 and 90–100 [35], suggesting a potential advantage for males, but detailed research is necessary. While many studies have shown a faster increase in disability among older adults with lower education, often from no ADL disability to having ADL disability [36,37], our study focused on a different scenario and found that older adults with lower education were at higher risk of worsening from mild to severe ADL disability. We found that older adults with no ADL disability in institutions had a lower risk of state deterioration, but the situation of those with ADL disability was not good. In institutions, older adults with middle ADL disability had a higher risk of state deterioration, and those with severe ADL disability had lower rates of state improvement. This may be because home care is China’s predominant mode of care [38]. Older adults are only institutionalized when care needs exceed family capacity, diminishing recovery prospects. China’s introduction of long-term care in the past two decades has led to persisting issues in institutional care, leaving the long-term care needs of older adults with ADL disability unmet [39]. A cohort study that focused on the income levels of older adults in the US and the UK showed that low-income individuals have a higher disability rate than high-income individuals [40]. This is similar to the current study, where older adults without pensions are more likely to transition from no ADL disability to mild ADL disability. Consistent with previous studies, co-morbidity impedes improvement in ADL disability state in older adults, but the current study demonstrated this pattern in only one transition scenario. Based on the analysis of influencing factors using the Markov model, we can observe the situation of multi-state transition. We found that among the older adults with chronic diseases, the influencing factors for state transition in those with different ADL disabilities were not the same, which helped us to identify the high-risk group of ADL disability and carry out targeted prevention and treatment for this group to reduce the occurrence of ADL disability or to slow down the development process of ADL disability.

The multi-state Markov model analyzed the complex mutual transitions between ADL disability states, but its generalizability is limited by data processing intricacy and the need for programming in specific studies [41]. Limited studies utilized a multi-state Markov model to examine ADL disability transitions in older Chinese adults. Research on transitional patterns between ADL states remains scarce in this population [31,42,43]. In addition, we focus on a specific group of older adults with chronic diseases because they not only have a high ADL disability but also have a high demand for healthcare and long-term care. Due to the lack of long-term care insurance coverage, older adults with chronic diseases in China may tend to overuse medical services, ultimately leading to an increase in medical expenses [44,45]. Therefore, we focused on the transition patterns and influencing factors between different ADL disability states in older adults with chronic diseases, aiming to improve their quality of life and reduce medical burden. Furthermore, as a city with a high degree of aging, Shanghai’s research results provide valuable experience and inspiration for other regions, which will help formulate more accurate health policies for older adults nationwide. Meanwhile, by comparing with other regions, differences in ADL disability among different areas can be revealed, providing a scientific basis for further improving the elderly health service system. These studies are of great significance for promoting the development of global elderly health.

Several advantages and limitations remain in the present study. The current study adopted long-term monitoring and the Markov model to accurately assess transitions between various ADL disability states, distinguishing itself from prior studies focusing on specific times or states. Our findings aid in predicting ADL disability transitions in older adults with chronic illnesses, informing strategies for early prevention and interventions to maintain health.

As for the limitations, the mortality rate had not been reported, which may potentially bias transition outcomes. Secondly, the sample of the current study is limited to Shanghai residents, which may affect generalizability. Thirdly, factors related to the COVID-19 pandemic were not considered, as the current study used data from 2014 to 2017.

In the next step of our research, we plan to use more representative samples to observe the transition patterns of ADL disability in older adults with each chronic disease to draw more accurate conclusions and better propose response measures. In addition, for public health issues, epidemics are an important factor, so we will compare the sample differences before and after the epidemic to explore it in depth.

## 5. Conclusions

In summary, it is common for older adults with chronic diseases to have their ADL disability state unchanged or improved as well as deteriorated. However, the state transition patterns in those with different ADL disability states are not the same, and the influencing factors for state transition in those with different ADL disability states are also not the same.

State transitions among older adults with chronic diseases vary by ADL disability severity, with those having mild ADL disability showing greater potential for improvement, while those with severe ADL disability face significant challenges, underscoring the need for targeted, proactive interventions, comprehensive care, and early prevention strategies to enhance quality of life and reduce healthcare costs.

The results of this study are beneficial for relevant departments to adopt different preventive and health measures for those experiencing different ADL disability states among older adults with chronic diseases to strengthen health management, improve their quality of life, promote the rational allocation of long-term nursing service resources, and effectively respond to the social care needs brought about by aging.

## Figures and Tables

**Table 1:** The baseline characteristics.

Characteristics	ADL Disability State			Total
	No ADL Disability	Mild ADL Disability	Severe ADL Disability	
**Age**				
60–69	1724	276	175	2175
70–79	1447	479	423	2349
80–89	1373	1296	1072	3741
90 or higher	108	387	331	826
**Sex**				
male	2135	906	740	3781
female	2517	1532	1261	5310
**Educational level**				
illiteracy	229	373	257	859
primary school	843	855	789	2487
middle school	1378	659	538	2575
high school	1585	393	279	2257
college or higher level	617	158	138	913
**Place of residence**				
home-based	4275	1043	147	5465
in institutions	377	1395	1854	3626
**Economic sources**				
with pensions	4549	2240	1838	8627
need allowances	103	198	163	464
**Chronic diseases grouping**				
non-co-morbidity	2949	946	509	4404
co-morbidity	1703	1492	1492	4687
Total	4652	2438	2001	9091

**Table 2:** ADL disability state transition in older adults with chronic diseases from 2014 to 2017.

Initial State	Transition Frequency			Transition Intensity			Transition Probability		
	The State After Transition			The State After Transition			The State After Transition		
	No ADL Disability	Mild ADL Disability	Severe ADL Disability	No ADL Disability	Mild ADL Disability	Severe ADL Disability	No ADL Disability	Mild ADL Disability	Severe ADL Disability
No ADL disability	6647	1556	176	−0.2767	0.2764	0.0003	0.8022	0.1758	0.0220
Mild ADL disability	1484	2538	810	0.4731	−0.6957	0.2226	0.3014	0.5452	0.1534
Severe ADL disability	118	366	3487	0.0068	0.1204	−0.1272	0.0258	0.0836	0.8905

**Table 3:** The multivariate analysis of ADL disability state transition in older adults with chronic diseases.

Variables	ADL Disability State Transition					
	1-2 HR (95% CI)	1-3 HR (95% CI)	2-1 HR (95% CI)	2-3 HR (95% CI)	3-1 HR (95% CI)	3-2 HR (95% CI)
**Age** (continuous variable)	1.09 (1.08–1.10) ^a^	1.41 (1.26–1.60) ^a^	0.98 (0.97–0.99) ^a^	1.01 (0.99–1.02)	1.03 (0.97–1.10)	1.01 (0.99–1.02)
**Sex** (male as control)						
female	1.05 (0.89–1.24)	2.31 (0.84–6.34)	0.95 (0.81–1.13)	0.78 (0.67–0.92) ^a^	3.69 (0.28–49.51)	0.65 (0.49–0.85) ^a^
**Educational level** (ordered categorical variable)	1.01 (0.93–1.10)	1.39 (0.86–2.24)	1.02 (0.94–1.10)	0.91 (0.84–0.99) ^a^	1.48 (0.84–2.60)	1.02 (0.90–1.14)
**Place of residence** (home-based as control)						
in institutions	0.77 (0.64–0.94) ^a^	0.01 (0.00–7.20)	0.13 (0.11–0.15)	1.39 (1.12–1.74) ^a^	0.04 (0.02–0.12) ^a^	0.16 (0.13–0.21) ^a^
**Economic sources** (with pensions as control)						
need allowances	1.20 (0.83–1.72)	4.65 (1.56–13.85) ^a^	0.65 (0.43–0.97) ^a^	1.23 (0.96–1.57)	1.30 (0.15–11.62)	1.27 (1.07–2.29) ^a^
**Chronic diseases grouping** (non-co-morbidity as control)						
co-morbidity	1.04 (0.88–1.22)	2.13 (0.82–5.54)	0.69 (0.58–0.81) ^a^	1.06 (0.91–1.24)	0.08 (0.00–1.49)	1.10 (0.75–1.63)

ADL disability state is represented by 1, 2, and 3: 1 represents no ADL disability, 2 represents mild ADL disability, and 3 represents severe ADL disability. Moreover, 1-2 represents a transition from no ADL disability to mild ADL disability, 1-3 represents a transition from no ADL disability to severe ADL disability, 2-1 represents a transition from mild ADL disability to no ADL disability, 2-3 represents a transition from mild ADL disability to severe ADL disability, 3-1 represents a transition from severe ADL disability to no ADL disability, 3-2 represents a transition from severe ADL disability to no mild disability. ^a^ *p* < 0.05; value 0.00 < 0.01.

## Data Availability

The data presented in this study are available on request from the corresponding author (Hansheng Ding) due to restrictions in Chinese population laws.

## Appendix A

**Table A1:** The measurement of ADL.

No.	Item
1	Whether assistance is needed to turn over in the prone position
2	Whether assistance is needed to sit up in the prone position
3	Whether assistance is needed to get up from a sitting position to a standing position
4	Whether assistance is needed to sit for at least 1 min
5	Whether assistance is needed to sit on a stool
6	Whether assistance is needed to maintain a bipedal stance for at least 10 s
7	Whether assistance is needed to walk (move) on level ground for about 45 m
8	Whether assistance is needed to walk (move) between rooms
9	Whether assistance is needed to balance the body
10	Whether assistance is needed to eat
11	Whether assistance is needed to brush/rinse the mouth
12	Whether assistance is needed to wash face and hands
13	Whether assistance is needed to comb the hair and apply simple makeup
14	Whether assistance is needed to put on/take off tops
15	Whether assistance is needed to put on/take off trousers
16	Whether assistance is needed to bathe (including shower and wipe) in general (not limited to 7 days)
17	Whether assistance is needed to use the toilet
18	Whether assistance is needed to trim fingernails in general (not limited to 7 days)
19	Is urinary incontinence present?
20	Is fecal incontinence present?

The answers to these questions were divided into five options:

Questions 1 to 18 were (1) no assistance is needed, (2) be able to complete with verbal guidance or care from others, (3) assistance from others is needed, but mainly complete the task on their own, (4) mainly reliant on assistance, with the older adults are just cooperating at most, and (5) complete assistance is needed or in more severe cases.

Question 19 was (1) can remain in control l every time, (2) can largely control, occasionally out of control, (3) out of control once a day or so, (4) largely out of control, occasional control, and (5) always out of control or more severe situations.

Question 20 was (1) can remain in control every time, (2) out of control less than once a month, (3) out of control about once a week, (4) largely out of control, occasional control, and (5) always out of control or worse every time.

According to the requirements of the SECUNA database, options one to five scored 1, 0.75, 0.5, 0.25, and 0, respectively.

**Table A2:** The univariate analysis of ADL disability state transition in older adults with chronic diseases.

Variables	ADL Disability State Transition					
	1-2 HR (95% CI)	1-3 HR (95% CI)	2-1 HR (95% CI)	2-3 HR (95% CI)	3-1 HR (95% CI)	3-2 HR (95% CI)
**Age** (continuous variable)	1.10 (1.09–1.11) ^a^	1.99 (1.50–2.64) ^a^	0.96 (0.95–0.97) ^a^	1.02 (1.01–1.03) ^a^	1.34 (1.09–1.65) ^a^	0.99 (0.98–1.01)
**Sex** (male as control)						
female	1.09 (0.98–1.21)	1.08 (0.00–5755)	0.74 (0.66–0.82) ^a^	0.94 (0.82–1.08)	1.70 (0.12–25.14)	0.77 (0.62–0.96) ^a^
**Educational level** (ordered categorical variable)	0.79 (0.75–0.83) ^a^	0.04 (0.01–0.69) ^a^	1.21 (1.15–1.27) ^a^	0.90 (0.85–0.96) ^a^	0.99 (0.51–1.96)	1.03 (0.93–1.13)
**Place of residence** (home-based as control)						
in institutions	2.13 (1.85–2.44) ^a^	2.03 (0.00–8470)	0.16 (0.14–0.19) ^a^	1.21 (1.04–1.40) ^a^	0.17 (0.01–2.67)	0.15 (0.12–0.19) ^a^
**Economic sources** (with pensions as control)						
need allowances	2.10 ^a^ (1.62–2.72) ^a^	1.07 (0.67–1.71)	0.35 (0.24–0.50) ^a^	1.50 (1.21–1.86) ^a^	3.86 (0.73–20.50)	1.37 (0.97–1.96)
**Chronic diseases grouping** (non-co-morbidity as control)						
co-morbidity	1.57 (1.40–1.75) ^a^	4.57 (0.00–2669)	0.55 (0.49–0.61) ^a^	1.25 (1.08–1.43) ^a^	0.44 (0.09–2.19)	0.92 (0.71–1.18)

ADL disability state is represented by 1, 2, and 3: 1 represents no ADL disability, 2 represents mild ADL disability, and 3 represents severe ADL disability. Moreover, 1-2 represents a transition from no ADL disability to mild ADL disability, 1-3 represents a transition from no ADL disability to severe ADL disability, 2-1 represents a transition from mild ADL disability to no ADL disability, 2-3 represents a transition from mild ADL disability to severe ADL disability, 3-1 represents a transition from severe ADL disability to no ADL disability, 3-2 represents a transition from severe ADL disability to no mild disability. ^a^ *p* < 0.05; value 0.00 < 0.01.
